# Visual guidance patterns and comprehension mechanisms in children’s nonlinear picture book reading: an eye-tracking study

**DOI:** 10.3389/fpsyg.2026.1755341

**Published:** 2026-06-17

**Authors:** Haoyu Sun, Xi Wang, Huangjia Chen

**Affiliations:** Graduate School of Techno Design, Kookmin University, Seongbuk-Gu, Seoul, Republic of Korea

**Keywords:** comprehension, eye-tracking, nonlinear narrative, picture book reading, visual attention

## Abstract

**Background:**

Nonlinear narrative picture books represent an emerging literacy form in children’s literature, yet their cognitive processing mechanisms remain understudied. This study investigated visual guidance patterns and comprehension mechanisms in 6-7-year-old children reading nonlinear narrative picture books using eye-tracking technology.

**Methods:**

Sixty-four children (72–95 months) read two Chinese nonlinear narrative picture books while their eye movements were recorded using Tobii Pro Spectrum (1,200 Hz). Four eye-tracking metrics were analyzed: fixation duration, path consistency index, cross-AOI scanning frequency, and image-first reading proportion. Comprehension was assessed through structured interviews evaluating factual understanding, inference, global coherence, and narrative structure understanding.

**Results:**

Children exhibited a predominant “image-first” pattern, with 58.6% of total fixation time on images versus 26.3% on text. However, mean fixation duration was longer for text (348.6 ms) than images (256.2 ms), indicating deeper text processing. Path consistency (r = 0.48, *p* < 0.001) and cross-AOI scanning (r = 0.45, *p* < 0.001) positively correlated with age. Eye movement measures explained an additional 26.3% variance in comprehension beyond baseline ability, with cross-AOI scanning as the strongest predictor (*β* = 0.45, *p* < 0.001). Cluster analysis identified three reader types: integrative readers achieved highest comprehension (M = 21.8), followed by text-dominant (M = 18.7) and image-dominant readers (M = 16.2).

**Conclusion:**

Findings extend text-image integration models to nonlinear contexts, supporting age-adaptive design principles and differentiated reading instruction strategies for children’s picture book reading.

## 1 Introduction

Reading picture books is an indispensable element of children’s linguistic skills, cognitive, and visual learning in early-childhood education. From the data in current literature, it is notable that reading of picture books greatly augments children’s comprehension of learning outcomes (Wang and Shao, 2025), and such social-inclined picture books assist children in developing their pro-social behavior skills too (Chen et al., 2025). During joint reading, children simultaneously apprehend all the multi-dimensional facets of learning (Breitfeld et al., 2021), and such a learning experience provided to children is an occasion fully arousing their cognitive stimulation. In this vein, the use of images and text in picture books is considered most suitable for multimodal learning because of the integration of images and text in a manner that stimulates the visual and linguistic cognitive channels of children’s brains (Chen and Huang, 2025), an idea that fits well with basic principles of dual coding theory. In recent years, together with advances in educational technology studies, designing a learning environment that is multimodal has become an increasingly focal point of research considerations (Aloizou et al., 2025), while research studies surrounding natural environment image relations in children’s cognitive development through picture books have become a hot research field in current research findings (Sun et al., 2025).

Modern children’s illustrated books experience a substantial revolution in the methodology of storytelling, as a new trend of non-linear storytelling appears. Nonlinear narrative picture books are defined as illustrated children’s books that deviate from conventional sequential storytelling by employing narrative techniques such as temporal reversals (flashbacks and flash-forwards), parallel multi-threaded storylines, spatial discontinuities, or reader-directed path choices, requiring readers to actively construct coherent meaning across non-sequential narrative elements. Unlike a traditional linear storytelling mode, these books use varying methods of storytelling such as reversal of time, simultaneous multi-threaded storytelling, or a leap in space, thereby presenting new challenges to children’s understanding. Narrative comprehension is a fundamental skill for children’s reading development, and strategic narrative interventions effectively improved auditory comprehension ability for special groups including children with autism (Spencer and Kirby, 2025). As a higher-level cognitive skill, the individual differences among preschoolers in inferential narrative comprehension are huge (Westerveld et al., 2021). Researchers emphasize that studies on narrative comprehension development need to pay more attention to increasing external validity and attending to relevant cognitive characteristics in children from diverse backgrounds (Burris and Brown, 2014). However, previous research about narrative comprehension has mainly focused on linear texts, and lack a systematical explorations on the cognitive processing mechanism of this kind of new emerging non-linear narrative form.

Eye-tracking technology allows a unique research window into understanding children’s reading cognition. Eye movement data can objectively record children’s attention and information processing in real time, superior to traditional reading tests. Takacs and Bus (2018) discovered that pictures in picture books support young children’s story comprehension with specific means. Simplified pictures in picture books enhance the attention and comprehension levels in novice readers (Eng et al., 2020). This illustrates that visual design features make a great difference in reading cognition. In Chinese contexts, the characteristics of children’s visual attention indicate the level of comprehension in reading picture books (Wang, 2020). There are also eye-tracking studies for special education; for instance, some have found that the design of a picture book affects the pattern of visual attention in autistic children (Lian et al., 2023). Although some attempts have been made by using eye-tracking technology in reading research with picture books, there is a shortage in non-linear narrative reading research up to now, not to mention a lack of systematic investigation of children in their critical transition period of 6–7 years old.

Multimodal learning theory is thus an important theoretical basis for understanding the cognitive mechanism of reading picture books. Computer-assisted learning, based on dual coding theory, demonstrates that learning outcomes are better when there is dual-channel encoding through the visual and linguistic pathways (Liu et al., 2020). The effect of multimodal input on language acquisition is influenced by cognitive load (Li et al., 2022), which suggests that researchers should focus on the role of working memory in text-image integration. The multimodal characteristics of digital programming devices can enhance children’s creative thinking (Tang et al., 2024), thus providing inspiration for multimodal integration in nonlinear narrative reading. However, most previous models of text-image integration are based on linear text construction and thus cannot fully explain the complication of nonlinear narrative reading; in addition, previous studies have paid inadequate attention to the developmental characteristics of children.

Previous research has established important foundations for understanding picture book reading. Eye-tracking studies have documented that children show strong visual preferences for illustrations (Takacs and Bus, 2018), that simplified visual designs enhance comprehension (Eng et al., 2020), and that visual attention patterns correlate with reading comprehension (Wang, 2020). However, these studies have primarily focused on linear narrative structures. The present study addresses these gaps by: (1) extending eye-tracking methodology to nonlinear narrative contexts, (2) employing age as a continuous variable to capture fine-grained developmental changes, and (3) identifying distinct reading pattern types that may inform differentiated instruction.

The need to investigate nonlinear narrative reading is grounded in both theoretical and practical considerations. Theoretically, existing models of text-image integration were developed primarily for linear multimedia materials and may not adequately explain how readers navigate and integrate information from non-sequential sources. Practically, children today encounter nonlinear information structures across various digital platforms—interactive e-books, websites with hyperlinks, and social media feeds—making the ability to process nonlinear content an increasingly essential literacy skill. The 6–7 year age range represents a particularly critical developmental period, as children transition from “learning to read” to “reading to learn.” Despite this importance, three fundamental limitations exist in current research: Theoretically, while cognitive models that integrate text and visual aspects have been developed, these models neglect the unique nonlinear storytelling characteristics. Methodologically, there is a lack of empirical studies that have explored children’s nonlinear reading processes using eye-tracking technology, and most developmental studies use cross-sectional age groups instead of continuous variables, which restricts detailed analysis of the developmental trajectories. Methodologically, nonlinear reading cognitive features during a critical transition period from “learning to read” to “reading as a learning tool” for 6-7-year-olds have not been given sufficient attention.

Specifically, this study addresses three research questions: (1) What are the characteristics of children’s visual attention allocation patterns when reading nonlinear narrative picture books? (2) How does age (measured continuously in months) relate to eye movement patterns and reading strategies? (3) To what extent do eye movement measures predict comprehension performance, and can distinct reading pattern types be identified?

This research uses eye-tracking to explore the patterns of visual guidance in nonlinear narrative picture book reading among 6-7-year-olds and their interlinkage with comprehension skills. By including age in months as a continuous variable, this study tries to reveal age effects on eye movement patterns, the critical role of cross-modal integration in understanding, and distinct reading pattern types. Theoretically, this extends the text-image integration model in nonlinear situations, proposes a cognitive mechanism model that applies to children’s nonlinear narrative reading, and precisely maps the cognitive changes in this critical developmental stage through continuous age variables. Empirically, these findings can help provide evidence for age-sensitive picture book design and stratified reading instruction that will benefit the cultivation of children’s ability to process nonlinear information in the digital age.

## 2 Materials and methods

### 2.1 Research design

**Figure 1:**
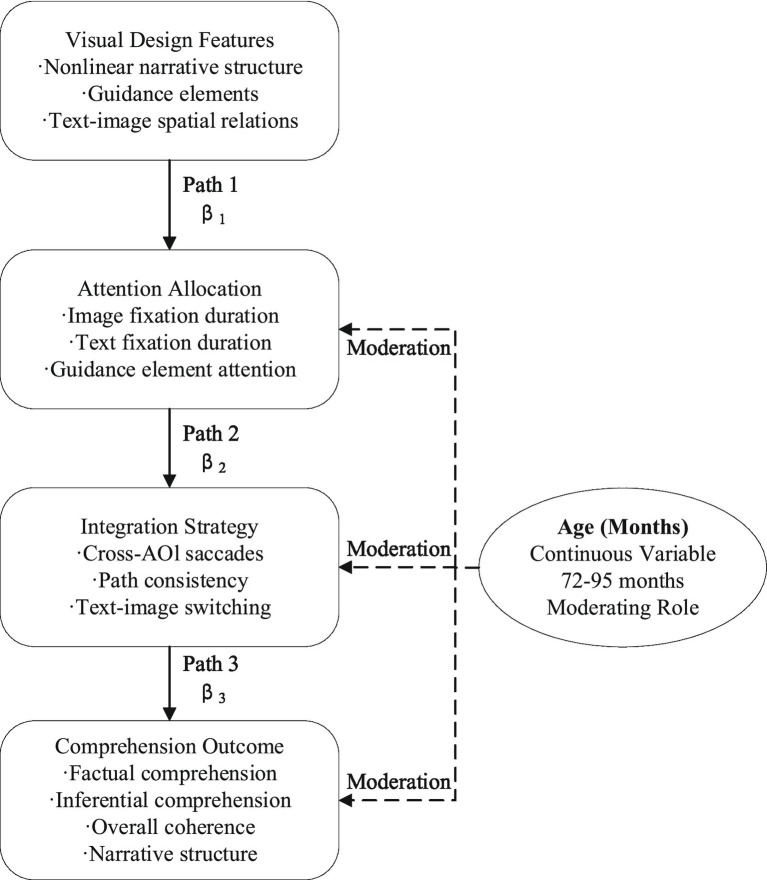
Conceptual model of nonlinear picture book reading.

The present study used a single-group observational design to examine the cognitive mechanism in reading nonlinear narrative picture books among children in the early school-age years. Eighty participants aged 6–7 (72–95 months) years old were recruited to read two identical nonlinear narrative picture books. Based on a correlational research paradigm, the relationships among eye movement metrics, age, reading ability, and comprehension performance were systematically investigated. Predictive variables included age, which was measured as a continuous variable in months, and standardized reading ability test scores; the outcome variables consisted of multiple eye movement indices such as fixation duration, saccade frequency, cross-interest area transition frequency, path consistency index, etc., and comprehension test scores in four dimensions covering factual understanding, inferential understanding, overall coherence, and narrative structure comprehension, with a total score of 30 points. The hypothesized theoretical framework is postulated (Figure 1) that visual design features affect children’s attention allocation patterns, which in turn affect the integration strategies and further affect comprehension results. Age in months acts as a continuous moderating variable in the whole cognitive processing, modulating the strength of the relations between the cognitive stages. Thus, integrating theories from visual cognition, multimodal integration, and developmental psychology, Figure 1 provides the conceptual basis that leads to a systematic investigation into the cognitive mechanism underlying nonlinear narrative reading among children. This study was approved by the Ethics Committee of Kookmin University (Approval No: 2024-EDU-056). Written informed consent forms were provided and signed by all guardians of the children before this study, and verbal consent from the children themselves was verbally obtained. The study fully complied with the Helsinki Declaration and the Guidelines for the Ethical Use of Children in Research.

### 2.2 Subject investigated

The sample size was calculated via correlation analysis using a moderate effect size of r = 0.30; the calculation showed that a total sample size of 64 participants would be needed. Given the expected 20% dropouts during data collection, the recruitment aimed at a sample of 80 children. Inclusion criteria were participants between 6.0 and 7.9 years who had acquired some basic independent reading skills in Chinese (Hua Yu) and with either normal or normally corrected vision. Exclusion criteria included children diagnosed as having reading disorder or ADHD; reporting serious problems with the acuity of their vision; and participants were missing eye movement data exceeded 30%. The final sample was evenly distributed by gender, comprising 32 boys and 32 girls, with a mean age of 78.24 months (6.52 years), SD = 6.96 months; youngest, 72 months, and oldest, 95 months of age. Given that these ages were reasonably evenly distributed in a monthly breakdown, 5–8 per age group, ages could be analyzed as a continuous variable. Reading performance (standardized test scores) averaged 72.5/100, SD = 12.3, reflecting considerable individual variation. These characteristics define a sample from which it would be reasonable to investigate effects of age on eye movement patterns.

### 2.3 Experimental materials

In this work, the authors carefully chose for experimental materials two Chinese picture books with salient nonlinear narrative features. In the selection, four key standards were followed: nonlinear narration is obvious with clear characteristics, such as temporal jumps or parallel multi-threaded storytelling; vocabulary should fit the target age group, with 800–1,500 words; visual guidance elements should be salient, such as arrows, numbered lines, color coding, or connecting lines; and the narrative language should be in Chinese. Picture Book A uses a narrative method combining chronological flashback with flashback. It contained 20 pages totaling around 1,300 words. To eliminate possible order effects, the order of presentation was varied. 40 subjects read from A to B, while 40 subjects read from B to A. Each of the picture books was coded in terms of its structural elements. Each page in the books was scored according to its narrative complexity ranging from 1 to 5 points. Various visual regulating elements in all books were coded, such as arrows, numbering of lines, use of color, as well as linking lines. Finally, all text-picture spatial relations in all books were coded as adjacent, separated, or overlapping.

**Figure 2:**
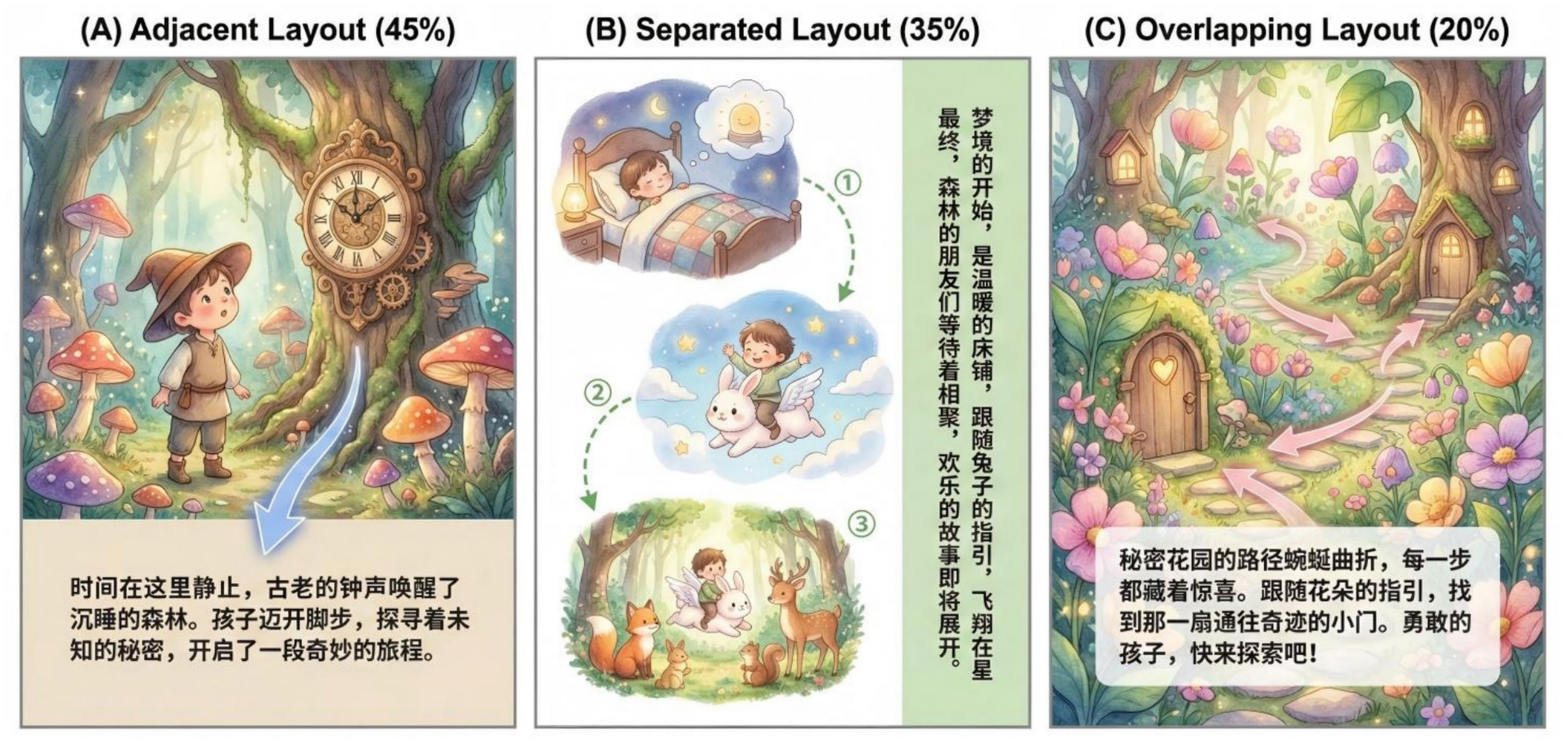
Representative page layouts in nonlinear narrative picture books. **(A)** Adjacent layout (45%). **(B)** Separated layout (35%). **(C)** Overlapping layout (20%).

Picture Book B employed parallel multi-threaded storytelling, containing 18 pages with approximately 1,100 words. Regarding page layout characteristics, illustrations occupied approximately 65–75% of each page area, while text regions occupied 20–30%, with the remaining 5–10% allocated to visual guidance elements. Illustrations were typically positioned in the upper or central portions of pages, with text placed below or alongside the images. On average, each page contained 50–80 Chinese characters. The spatial arrangement of text and images varied across pages: approximately 45% of pages featured adjacent text-image layouts (text directly below or beside images), 35% featured separated layouts (text and images in distinct page regions), and 20% featured overlapping layouts (text embedded within or overlaying images). Figure 2 presents representative page examples illustrating these layout patterns and AOI definitions.

The selection of 20-page and 18-page picture books was based on several considerations. First, commercially available nonlinear narrative picture books for this age group typically range from 15–24 pages, and our materials fall within this standard range. Second, pilot testing with 8 children (not included in the final sample) indicated that children could complete reading both books within the allocated time without significant fatigue. Third, the total reading time (8–10 min per book) aligns with attention span expectations for 6-7-year-olds. Finally, using full-length picture books rather than truncated excerpts enhances ecological validity, as children encounter complete narratives in natural reading contexts.

### 2.4 Eye tracker devices and metrics

In this experiment, data about children’s reading process was collected by an eye-tracker system known as Tobii Pro Spectrum, whose sampling rate is 1,200 Hz and is capable of tracking fast eye movements accurately. Eye movement data were recorded and analyzed using Tobii Pro Lab software (version 1.194), which was used for stimulus presentation, real-time data recording, areas of interest (AOI) definition, and extraction of fixation and saccade metrics. Also, in this experiment, materials’ presentation was done at the center of a 24-inch screen with a screen resolution of 1920 × 1,080 pixels. Moreover, in this research, viewing distance was set at 60–65 cm between a child and a screen. Three categories of eye movement metrics, fixation, saccade, and path metrics, were extracted in this study to comprehensively reflect the reading cognitive process of children. Fixation metrics include total fixation duration, average fixation duration, and fixation frequency, reflecting the depth of cognitive processing in certain regions. Longer fixation durations are generally associated with deeper cognitive processing and greater processing difficulty (Mézière et al., 2023). Saccade metrics include saccade frequency, average saccade amplitude, and cross-interest-region saccade frequency. The latter is regarded as one of the most important behavioral indices for cross-modal integration of text and image (Alemdag and Cagiltay, 2018). Path metrics include path consistency index, interest-region access sequence, and revisitation frequency, reflecting the degree of adherence to designed reading paths and the flexibility of their reading strategies. In the current study, pages of picture books were divided into four kinds of interest regions for detailed analysis, including Text Interest Region (all textual content), Image Interest Region (all illustration content), Guiding Elements Interest Region (specific guiding elements, such as arrows and numbers), and Narrative Key Points Interest Region (core information in a story). This systematic interest region classification offers a quantitative foundation for analyzing and understanding the pattern of attention allocation as well as nonlinear narrative cross-modal integration strategy in children.

### 2.5 Experimental procedure

Data collection involved a quiet lab setting and took a total of 35 min. The experimental setup, including eye-tracker positioning and participant seating arrangement, is illustrated in Supplementary Figure S1. After a 5-min introduction to the experimental setting, children were allowed to get acquainted with it as well as with themselves. Calibration of eye tracking was performed using a 5-point calibration procedure to ensure that precision was achieved in data collection, which took 3–5 min. Calibration was accepted when the average accuracy was below 0.5° of visual angle; otherwise, recalibration was performed. Subsequently, an activity of 3 min was done, allowing children to become conversant with reading. Finally, reading of two pictorial books worth 8–10 min gave children a chance to read at will and as often as they wanted. In between was a 2-min pause, helping to avert fatigue that could hamper data validity. To further assess potential fatigue effects, we compared eye movement metrics (total fixation duration, mean fixation duration, cross-AOI scanning frequency) and comprehension scores between the first and second books read. Paired t-tests revealed no significant differences in any metric between Book 1 and Book 2 (all ps > 0.15), suggesting that the 2-min break and overall session design effectively mitigated fatigue effects. Additionally, research assistants monitored children for signs of fatigue (e.g., restlessness, decreased attention) throughout the session, and no sessions were terminated early due to participant fatigue. Simple directions were provided in respect of reading, such as, “Now, read as you would, and we will ask you a few questions later.” After reading both books, a 10-min comprehension test was carried out through structured interviews. This followed a 2-min thank you session where rewards in form of small presents were given to terminate the experiment. During this experiment, research assistants were present to ensure that all children felt at ease and adhered to all protocols.

### 2.6 Measurement of comprehension ability

An assessment rubric was developed involving four components: factual, inferential reasoning, overall coherence, and narrative structure. In fact, the factual component, which checks children’s capacity for recalling and remembering key details of a story, is marked by 6 questions that are worth 1 point. By contrast, the cognitive load is higher in terms of inferential reasoning, so it constitutes a total of 6 questions that are worth 2 points and checks children’s capacities for inferring from text and pictorial clues. Coherence aspect of the total score is an assessment of children’s ability to link discrete elements of a story together in meaningful ways and was comprised of 3 questions with 3 points for each question. Narrative structure aspect was comprised of 2 questions addressing metacognitive understanding of non-linear narrative structures with 1.5 points for each question. Response was done through structured oral interviews. The answers provided by participants were scored through a double-blind process in order to make it objective. Answers from participants were scored by two independent raters while they were blinded to participants’ ages as well as their eye tracking data. Interrater reliability was calculated through Cohen’s Kappa, and a score above 0.80 is a good indication of reliability. Any inconsistencies between scores of raters were addressed through consensus or independent raters.

### 2.7 Data processing and analysis methods

Eyetracker data was preprocessed in accordance with strict quality control standards in this study. In fixation point filtering, the configuration of the filtering criteria was that of >80 ms and <1,200 ms. Data points with values above 30% missing per page were removed, and data from all participants with total missing data points above 30% were also removed. Outliers were defined based on the mean ± 3 standard deviations and then processed by Winsorization to retain the sample size. Statistical analysis was performed according to a multi-level analytical strategy to answer each research question comprehensively. Descriptive statistics consisted of mean values, standard deviations, and ranges for all variables. Correlation analysis was performed using Pearson’s correlation coefficients between eye movement measures and comprehension scores/age. Regression analyses were structured as follows: simple linear regression was conducted to determine the predictive effect of age on eye movement measures; hierarchical regression analyses built up two regression models that predicted comprehension scores: Model 1 (controlling for age and reading ability); and Model 2 consisted of Model 1 and added eye movement measures to determine incremental explanatory power of eye movement measures over age and reading ability; moderation-effect analysis of age was used for investigating its moderating role through interaction terms. Cluster analysis: K-means method was used to identify distinct reading pattern types based on the standardized eye movement measures. Effect sizes were reported, including correlation coefficients, r, coefficients of determination, R^2^, standardized regression coefficients, *β*, and Cohen’s d. A significance level was set at *α* = 0.05, and Bonferroni correction for multiple comparisons was applied. Analyses of eye movement data were made by Tobii Studio 3.4, statistical analyses by SPSS 27.0 or R 4.3, and visualizations by the ggplot2 package in R.

## 3 Results

### 3.1 Descriptive statistics

Eventually, the current study obtained valid data from 64 children aged 6–7 years. Among the original 80 participants, 16 were excluded because of poor data quality-more than 30% data missing or severe eye-tracking failures. Below, descriptive statistics of retained samples and key variables are presented in Table 1. The children’s age ranged from 72 to 95 months, with an average of 78.24 months (standard deviation: 6.96 months), and a balanced gender distribution (32 boys vs. 32 girls). Results of the reading ability test showed a large individual difference in scores, with an average of 72.5 out of 100, a standard deviation of 12.3 points, thereby ensuring that the sample is diverse in reading skills. The data obtained from eye tracking was of high quality, with an average of 93.8% data completeness (ranging from 87.5 to 98.6%). This data is accurate and suitable for further analysis. In the eye tracking experiment, fixation duration of children’s total fixation while nonlinear picture books were being read showed an average of 234.6 s with a standard deviation of 45.3 s, which implied large individual differences in the data. Average fixation duration was 286.4 milliseconds, while its standard deviation was 52.1 milliseconds, which is normal in children’s reading studies. On average, the scanning rate of video was 148.2 per minute with a standard deviation of 28.6 per minute, showing frequent shifting of visual fixation in nonlinear narrative processing. For a major measure of text-image integration, as a variable that conveyed large strategic differences, the scanning rate of video based on cross-interest area was 42.8 per minute with a standard deviation of 12.4. The path consistency index was on average 0.64 with a standard deviation of 0.18, showing that there was a moderate tendency to follow designed reading paths with big individual differences among children. The comprehension test total score averaged 18.4 points out of 30 with a standard deviation of 4.6; fact comprehension was the highest at 78.3%, followed by 65.4% for inference comprehension, and 58.7% for overall coherence, while narrative structure was lowest at 52.5%. This would suggest that nonlinear narrative texts are challenging for children’s high-order cognitive abilities.

**Table 1:** Descriptive statistics of sample characteristics and main variables.

Variable	M	SD	Min	Max	Range
Sample characteristics					
Age (months)	78.24	6.96	72	95	23
Reading ability (0–100)	72.5	12.3	45.2	96.8	51.6
Data completeness (%)	93.8	3.2	87.5	98.6	11.1
Eye movement metrics					
Total fixation duration (s)	234.6	45.3	152.8	358.2	205.4
Mean fixation duration (ms)	286.4	52.1	198.6	412.3	213.7
Saccade frequency (per min)	148.2	28.6	95.4	218.6	123.2
Cross-AOI saccades (per min)	42.8	12.4	18.2	72.6	54.4
Path consistency index (0–1)	0.64	0.18	0.28	0.95	0.67
Comprehension test scores					
Total score (0–30)	18.4	4.6	9.2	28.5	19.3
Factual comprehension (0–6)	4.7	0.9	2.5	6.0	3.5
Inferential comprehension (0–12)	7.8	2.1	3.5	11.5	8.0
Overall coherence (0–9)	5.3	1.6	2.0	8.5	6.5
Narrative structure (0–3)	1.6	0.7	0.0	3.0	3.0

N = 64 (32 boys, 32 girls). Sixteen participants were excluded due to excessive missing eye movement data (>30%). AOI = area of interest.

### 3.2 General characteristics of the visual guidance path

**Figure 3:**
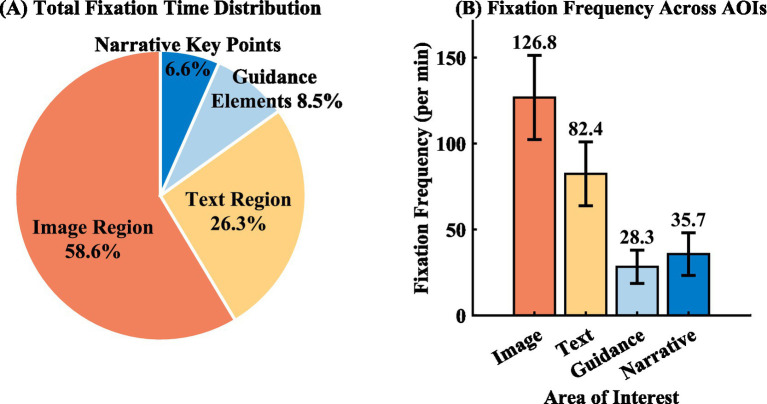
Visual attention allocation patterns. **(A)** Total fixation time distribution. **(B)** Fixation frequency across AOIs.

This study systematically analyzed children’s visual attention allocation patterns during nonlinear picture book reading. Table 2 reflects the statistical result of eye movement indicators in each interest area. The results showed that an obvious “image-first” reading pattern, with the proportion of total fixation time for visual interest areas at 58.6% (std. 12.4%), was significantly higher than that for textual interest areas (26.3%, std. 8.7%). Guiding element areas and narrative key points areas took up 8.5 and 6.6%, respectively, indicating very limited attention to the guiding symbol. Further, children had a pronounced inclination for image areas in their first fixation (82.5%), followed by text (12.8%) and guiding elements (4.7%) (Figure 3A), indicating an important tendency toward the image area and reflecting children’s special cognitive characteristics at this age, which is influenced by the strong appeal of visual information. Average fixation duration is a variable that registers subtle differences in cognitive processing, where textual interest areas have recorded a longest fixation duration of 348.6 ms, std. 78.4 ms, which is significantly higher than image areas at 256.2 ms, std. 52.1 ms, and guiding elements areas at 198.7 ms, std. 45.3 ms. Although children spend more time on images, it can be seen that they process textual information deeper per fixation. The annotation frequency statistics further verified this observation: in image regions, children had the largest number of annotations, with up to 126.8 annotations per minute (std. 24.5), followed by 82.4 for text regions (std. 18.6) and only 28.3 for guiding elements, std. 9.7, as illustrated in Figure 3B. Noticeably, the cross-interest area conversion pattern revealed that image-text transition occupied 67.4% of all cross-region scans, while the transition with guiding elements occupied only 18.3%. It is obvious that children mostly conduct integration between text and images, with the explicit guiding symbols not being sufficiently used.

**Table 2:** Eye movement metrics across different areas of interest.

Area of interest	Total fixation time (%)	Mean fixation duration (ms)	Fixation frequency (per min)	First fixation preference (%)
Image region	58.6 (12.4)	256.2 (52.1)	126.8 (24.5)	82.5
Text region	26.3 (8.7)	348.6 (78.4)	82.4 (18.6)	12.8
Guidance elements	8.5 (4.2)	198.7 (45.3)	28.3 (9.7)	4.7
Narrative key points	6.6 (3.8)	312.4 (68.2)	35.7 (12.4)	−

Values are presented as M (SD). *N* = 64. Total fixation time percentages sum to 100%. Narrative key points overlap with image and text regions, thus no first fixation preference data.

### 3.3 Age affects the continuity of eye movement patterns

**Figure 4:**
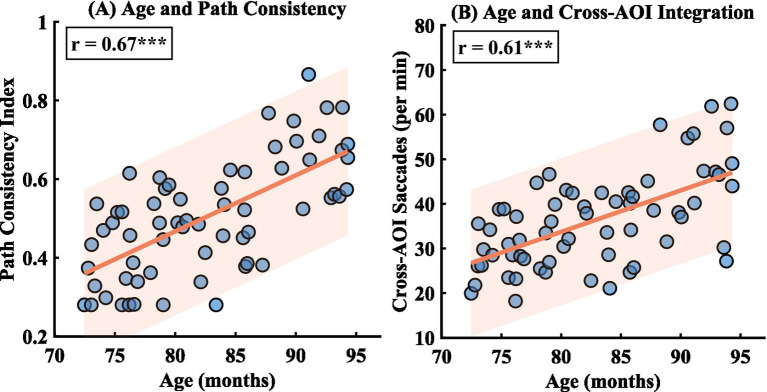
Developmental changes in reading strategies. **(A)** Age and path consistency. **(B)** Age and cross-AOI integration.

In this study, age in months was treated as a continuous variable in an effort to capture the precise developmental trajectory in children’s eye movement patterns. The correlation analysis between age and key eye movement indices is shown in Table 3. The results showed that the age in months was positively correlated with the path consistency index (r = 0.48, *p* < 0.001), suggesting that the tendency of children to follow the designed reading paths increases progressively with their age, as demonstrated in Figure 4A. The scatter plot indicated a clear linear growth trend, where the path consistency index averaged 0.56 (SD = 0.16) for children aged 72–84 months but increased to 0.72 (SD = 0.14) for the age group of 85–95 months, with a 28.6% increase. This showed that children’s visual navigation ability possessed a characteristic of continuous development. In addition, a significant positive relationship was found in age in months and the scanning of cross-interest-area, as shown in Figure 4B, with a correlation coefficient of r = 0.45, *p* < 0.001, illustrating that older children have a greater level of initiative in text-image integration. In particular, the average scanning rate was 36.2 per minute (SD = 10.8) in children aged 72–78 months, 42.6 per minute (SD = 11.5) in children aged 79–84 months, and 49.8 per minute (SD = 12.6) in children aged 85–95 months, increasing step by step. No significant correlation, by contrast, was found in terms of total fixation duration (r = −0.12, *p* = 0.342) or average fixation duration (r = 0.08, *p* = 0.531) with age in months, suggesting that baseline fixation metrics remain relatively stable during this developmental stage. More importantly, age in months was moderately associated with the total comprehension test scores (r = 0.52, *p* < 0.001), which was mediated by path consistency and cross-AOI scanning to a certain extent. Therefore, the age of 6–7 years constitutes a critical period for the development of reading strategies in children, and cognitive improvement within this age range mainly embodies itself in terms of better efficiency in visual navigation and proactive integration of text and image rather than in increased fixation duration.

**Table 3:** Correlations between age (months) and eye movement metrics.

Eye movement metric	r	95% CI	*p*	Interpretation
Path consistency index	0.48***	[0.26, 0.65]	<0.001	Strong positive correlation
Cross-AOI saccade frequency	0.45***	[0.23, 0.63]	<0.001	Strong positive correlation
Total fixation duration	−0.12	[−0.36, 0.14]	0.342	No significant correlation
Mean fixation duration	0.08	[−0.17, 0.32]	0.531	No significant correlation
Saccade frequency	0.31*	[0.07, 0.52]	0.014	Moderate positive correlation
Fixation on guidance elements (%)	0.38**	[0.15, 0.57]	0.002	Moderate positive correlation
Comprehension total score	0.52***	[0.31, 0.68]	<0.001	Strong positive correlation

*N* = 64. CI = Confidence Interval. *p < 0.05, **p < 0.01, ***p < 0.001.

### 3.4 The predictive role of eye movement indicators on understanding

**Figure 5:**
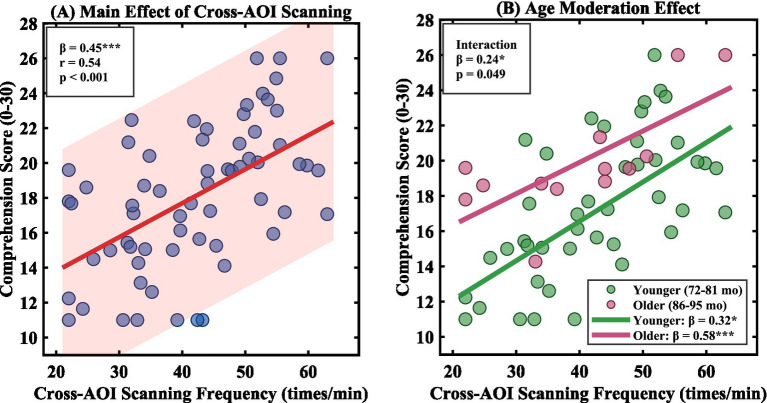
The predictive role of cross-AOI scanning frequency on comprehension performance. **(A)** Main effect of cross-AOI scanning. **(B)** Age moderation effect.

This study conducted hierarchical regression analysis to investigate the incremental predictive value of eye movement measures for comprehension performance. Table 4 displays full results of regression analysis. Within the baseline model that included control of reading ability as well as age (model 1), it was determined that reading ability and age explained 32.1% of comprehension variance, whereby R^2^ = 0.321, *F* (2, 61) = 14.42, *p* < 0.001. In terms of beta values, it was found that while age was a significant predictor, *β* = 0.38 (*p* < 0.001), reading ability was also a significant predictor of comprehension, *β* = 0.31 (*p* = 0.006). By adding all four eye movement variables in Model 2, there was an improvement in explanatory power to 58.4%, R^2^ = 0.584, with an incremental value of 26.3%, ΔR^2^ = 0.263, ΔF (4, 57) = 9.87, *p* < 0.001. This indicates that eye movement variables make a unique contribution to comprehension above baseline skills. Among those variables, the cross-interest region video scanning rate has the highest predictive power, *β* = 0.45, *p* < 0.001, which suggests that proactive integration of text and image is the main process involved in nonlinear comprehension of a narrative text. On the other hand, a path consistency index has a large effect as a predictor, *β* = 0.28, *p* = 0.004, because it facilitates scanning based on visual guidance for understanding. In terms of total fixation duration, it has a weak effect as a predictor, *β* = −0.18, *p* = 0.082, while average fixation duration lacks a significant effect, β = 0.11, *p* = 0.256. On examination of moderating variables involving age, it was found that it was a significant moderator in predicting comprehension based on scanning across AOI, interaction term β = 0.24, *p* = 0.049. As shown in Figure 5, older children (85 ~ 95 months) present stronger predictive power of cross-AOI scanning for comprehension, simple slope β = 0.58, *p* < 0.001, whereas younger children (72 ~ 78 months) show a relatively weaker effect, simple slope β = 0.32, *p* = 0.026. These results suggest that when children become older cognitively, they are more capable of exploiting text-image integration strategies to improve comprehension, reflecting interaction between eye movement patterns and cognitive development.

**Table 4:** Hierarchical regression analysis predicting comprehension performance.

Predictor	Model 1			Model 2		
	B	SE	β	B	SE	β
Control variables						
Age (months)	0.24	0.07	0.38***	0.15	0.06	0.24*
Reading ability	0.11	0.04	0.31**	0.09	0.03	0.25**
Eye movement metrics						
Cross-AOI saccades				0.17	0.04	0.45***
Path consistency index				7.23	2.38	0.28**
Total fixation duration				−0.01	0.01	−0.18
Mean fixation duration				0.01	0.01	0.11
Model statistics						
R^2^	0.321			0.584		
Adjusted R^2^	0.299			0.541		
F	14.42***			13.68***		
ΔR^2^				0.263***		
ΔF				9.87***		

*N* = 64. B = unstandardized regression coefficient; SE = standard error; β = standardized regression coefficient. Model 1: Control variables only. Model 2: Control variables + eye movement metrics. *p < 0.05, **p < 0.01, ***p < 0.001.

### 3.5 Individual reading mode differences

**Figure 6:**
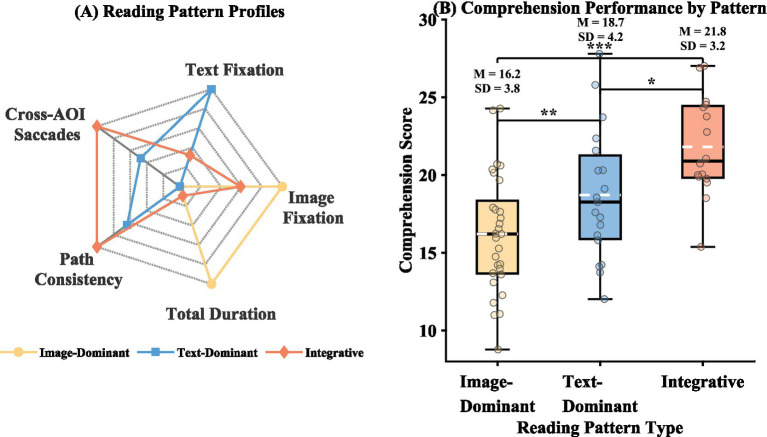
Individual differences in reading patterns. **(A)** Reading pattern profiles. **(B)** Comprehension performance by pattern.

This study used K-means clustering analysis to identify individual differences in children’s non-linear picture book reading, using the standardized eye movement metric of total fixation duration, average fixation duration, cross-AOI scanning frequency, and path consistency index for clustering. The contour coefficient analysis suggested that three clusters were optimal (coefficient = 0.58, significantly better than the two clusters’ 0.41 and four clusters’ 0.47). Three different reading patterns emerged from the clustering results, and their eye movement characteristics and comprehension performance are illustrated in Table 5. The image-dominant type showed the highest fixation duration in image regions (M = 68.4%, SD = 8.2%), with the lowest cross-AOI scanning frequency (M = 32.5 times/min, SD = 8.6), and the weakest path consistency index (M = 0.54, SD = 0.16), as Figure 6A reveals. These children tended to dwell too much in image areas with inactive text-image integration, leading to significantly lower comprehension scores compared to the other two groups (M = 16.2 points, SD = 3.8). In the text-dominant type, a reversed pattern was found: 54.6% of fixations in text (SD = 9.4%), a moderate frequency of cross-AOI scanning (M = 42.1 times/min, SD = 10.2), good path consistency (M = 0.68, SD = 0.15), and a moderate level of comprehension scores (M = 18.7 points, SD = 4.2). The integrated group showed the most effective reading strategy, with relatively even distribution of attention to images at 57.8% and text at 30.2%. Children in the integrated group demonstrated the highest cross-AOI scanning frequency (M = 52.8 scans/min, SD = 9.8) and the strongest path consistency index (M = 0.76, SD = 0.12), as Figure 6B depicts, whose comprehension scores were significantly higher than those of the image-dominant group, with M = 21.8 points, SD = 3.2, Cohen’s d = 1.32. One-way ANOVA revealed a significant difference among the three groups on comprehension scores: *F* (2, 61) = 15.84, *p* < 0.001, η^2^ = 0.34. The subsequent post-hoc test revealed that the integrated group outperformed both the image-dominant (*p* < 0.001) and text-dominant (*p* < 0.001) groups.

**Table 5:** Eye movement characteristics and comprehension performance across three reading patterns.

Variable	Image-dominant (*n* = 29)	Text-dominant (*n* = 19)	Integrative (*n* = 16)	F	*p*	η^2^
Eye movement metrics						
Image fixation time (%)	68.4 (8.2)	42.3 (7.6)	57.8 (6.4)	62.34	<0.001	0.67
Text fixation time (%)	18.6 (5.4)	54.6 (9.4)	30.2 (5.8)	98.47	<0.001	0.76
Cross-AOI saccades (per min)	32.5 (8.6)	42.1 (10.2)	52.8 (9.8)	22.16	<0.001	0.42
Path consistency index (0–1)	0.54 (0.16)	0.68 (0.15)	0.76 (0.12)	11.28	<0.001	0.27
Total fixation duration (s)	248.6 (42.3)	226.4 (38.7)	228.5 (41.2)	1.85	0.165	0.06
Comprehension performance						
Total score (0–30)	16.2 (3.8)ᵃ	18.7 (4.2)ᵇ	21.8 (3.2)ᶜ	15.84	<0.001	0.34
Factual comprehension (0–6)	4.2 (0.9)	4.6 (0.8)	5.1 (0.7)	6.42	0.003	0.17
Inferential comprehension (0–12)	6.8 (2.3)ᵃ	7.9 (2.0)ᵇ	9.2 (1.8)ᶜ	7.89	0.001	0.21
Overall coherence (0–9)	4.5 (1.7)ᵃ	5.4 (1.5)ᵇ	6.3 (1.3)ᶜ	8.15	0.001	0.21
Narrative structure (0–3)	1.2 (0.7)ᵃ	1.6 (0.6)ᵃᵇ	2.1 (0.5)ᵇ	10.27	<0.001	0.25
Demographics						
Age (months)	77.2 (7.1)	78.6 (6.5)	80.1 (6.8)	0.98	0.381	0.03
Reading ability (0–100)	69.4 (12.8)	73.8 (11.6)	75.2 (12.1)	1.23	0.299	0.04

Values are presented as M (SD). *N* = 64. Different superscript letters (^ᵃ, ᵇ, ᶜ^) indicate significant differences between groups based on Tukey’s HSD post-hoc tests (*p* < 0.05). F-values are from one-way ANOVA. Η^2^ = effect size (partial eta-squared).

## 4 Discussion

This study employed eye-tracking technology to systematically investigate the pattern of visual guidance and the comprehension mechanism in nonlinear picture book reading among 6-7-year-old children and obtained several important findings. The “image priming” phenomenon detected here is also highly consistent with the conclusion of previous studies on picture book reading. Roy-Charland et al. (2007) also noted that preschoolers showed strong image preference in shared reading studies, a pattern reflecting the universal characteristics of visual cognitive development in this period. However, in this study, further verification indicated that although the duration of the child’s fixation in image regions was longer, the average fixation time on text areas was significantly higher. A dissociation between fixation duration and fixation frequency suggests deeper cognitive processing. Research by An et al. (2017) on reading pattern differences between adults and children offers an explanation: it is possible that children need more cognitive effort to extract information from texts, while images achieve it through rapid multiple saccades. Design research on e-books also supports the influence of visual complexity on children’s attention. According to Liao et al. (2020), simplified illustration designs increased the attention and comprehension levels of children in kindergartens. It may be that overcomplex visual presentations interfere with the effective allocation of attention.

It is important to acknowledge that the eye movement patterns observed in this study reflect an interaction between children’s cognitive processing strategies and page-specific visual features. The “image-first” pattern, for instance, may be partially driven by the visual salience of illustrations, which typically occupied larger page areas and were positioned in prominent locations. However, several findings suggest that the observed patterns also reflect genuine cognitive strategies. First, the significant correlation between cross-AOI scanning frequency and comprehension scores suggests that text-image integration behaviors, rather than mere visual salience responses, contribute to understanding. Second, the developmental increases in path consistency and cross-AOI scanning with age indicate progressive strategy refinement that goes beyond stimulus-driven responses. Third, the identification of distinct reader types with different comprehension outcomes suggests individual differences in strategic reading approaches. Nevertheless, future studies should consider employing controlled stimuli with systematically varied layout features to disentangle stimulus-driven and strategy-driven components of visual attention.

Limitations in the capacity of working memory may thus be closely associated with the differences in cognitive processing mechanisms. Candal and de Avila (2025) demonstrated that phonological working memory and language processing speed play an important role in inferential reading comprehension. Nonlinear narratives, due to their complicated structure, may require higher working memory when compared to linear ones. Intervention studies have also confirmed the role of working memory in reading development. Okur and Aksoy (2025) showed that working memory intervention significantly improved reading performance among students with special learning disabilities, which indicated that working memory training may thus be an effective way to improve reading. This study found a close relationship between cross-interest area scanning frequency and comprehension performance. The nature of such frequent visual transitions likely requires extra working memory resources, since children need to maintain and integrate information from different modalities in short-term memory. Nouwens et al. (2017) emphasized that working memory exerts its influence on children’s reading comprehension in a domain-specific manner. Storage and processing functions play different roles during comprehension. Therefore, it provides a theoretical basis for explaining individual differences in nonlinear narrative reading.

In the present study, age as a continuous variable is included in the analytical framework, reflecting progressive development. A linear growth trend in the Path Consistency Index and video scanning frequency across interest areas proved consistent with increasing age. This agrees with broader research on cognitive development since Berger et al. (2025), in one of the largest intervention studies to date, have replicated sustained effects of working memory training on the cognitive and non-cognitive skills of children, thereby underlining the plasticity and continuity of cognitive development. On the basis of the close link between executive functions and reading fluency, the literature further underlines the role of cognitive control in reading comprehension. According to Colombino et al. (2025), executive functions indirectly affect comprehension performance by regulating reading fluency; this mechanism might be extended to the context of nonlinear narrative reading. Of relevance is the fact that the promoting effect of cognitive stimulation items on reading comprehension has been verified in repeated studies. Reina-Reina et al. (2023) conducted a cognitive stimulation program resulting in significant improvement in reading comprehension in elementary school children, thus providing an empirical basis for designing differentiated intervention strategies in consideration of children with different reading pattern types.

In this study, visual attention control development is manifested as the older the children, the better their facility in following designed paths and in proactively integrating text and image. The research by Moyano et al. (2022) studied the association between the visual attention control development in preschoolers and temperament/family environment, demonstrating a multi-factor mechanism that influenced the development of attention, which probably meant that individual differences in reading patterns can be moderated by multiple factors other than cognitive elements. The importance of visuospatial attention to reading development has been consistently supported through meta-analysis. Gavril et al. (2021) systematically reviewed the relationship between visuospatial attention and reading development and confirmed that attention control ability is stably associated with reading performance. Based on neurocognitive development, the dynamic framework of individual differences provides a new theoretical perspective to understand the development of reading ability in children. The dynamic framework of reading development put forward by Bonte and Brem (2024) emphasizes individual differences in learning potential and its neural basis, which coincides with the three kinds of reading patterns found in this study.

This present study found that the video scanning rate across interest domains, as a behavioral indicator of text-image integration, had the strongest predictive power on comprehension. Empirical evidence here supports the application of multimedia learning theory in nonlinear narrative contexts. Mayer (2024), while reviewing cognitive theories of multimedia learning, noted that dual-channel processing and active integration are necessary. This study’s results not only verify this theoretical background but also identify the developmental character of integration as a whole. Studies in technology-enhanced storybooks are also related to explaining the complex effect of the elements of multimedia learning on learning results. In a meta-analytic study, Takacs et al. (2015) concluded that multimedia and interactive elements have a beneficial effect but also entail a risk that too much visual exposure might distract children without supplementing their understanding. The balanced allocation of attention and active strategies for text-image integration found in the integrated readers in this study are characteristics typical of effective multimedia learning.

The use of reading interventions in realistic conditions presents a means of implementing the results in practice. Lingwood et al. (2020) carried out a review of evidence for shared reading interventions with preschool children and their families and showed that structured reading sessions led to a positive effect on early literacy skills. This is informative, as targeted interventions based on these findings would be of practical value. Apart from this, the content features of reading materials are of relevance for their consequences for children’s behavior and cognition. Yao and Enright (2020) found that moral stories affected sharing behaviors in kindergarten children, and it may thus be proposed that the thematic content of nonlinear narratives modulates comprehension effectiveness through the effects of emotional engagement and motivational levels.

Although this study obtained several important results, it still has some limitations. First, the observed “image-first” pattern and greater fixation time on visual elements may be partially influenced by page layout characteristics rather than purely reflecting cognitive processing preferences. Since illustrations were consistently more prominent, larger, and positioned in visually salient areas (upper or central regions of pages), this could naturally draw children’s gaze first and sustain attention on images. Future studies should consider controlling for layout variables or employing eye-tracking measures that account for stimulus salience. Second, the varied nonlinear layouts across pages, including different visual cues and structural elements, may have elicited page-specific eye movement patterns that reflect design features rather than consistent reading strategies. Pages with explicit directional arrows may prompt more predictable fixation sequences than pages without such cues. While this variability reflects the naturalistic characteristics of commercially available nonlinear picture books, it introduces heterogeneity that may complicate interpretation. To address this, we analyzed eye movement metrics aggregated across all pages, which captures general tendencies while reducing the influence of any single page’s unique features. Future studies could employ more controlled stimuli with systematically varied guidance elements to isolate the effects of specific design features. Third, the sample was primarily urban and did not include rural or multicultural children, which might have some implications for the generalization of the findings. Because of the cross-sectional design, no causal relation could be established; hence, a follow-up study should be conducted using longitudinal tracking or intervention experiments to further explicate the developmental trajectory and the causality issue. Cognitive variables, such as working memory capacity and executive functions, known to be important factors in reading comprehension, were not measured in the current study. Future studies should also examine the individual effect of varied nonlinear narrative structures on children’s reading cognition. Moreover, it is worth comparing the differences in cognitive processing of digital reading and reading a picture book in a paper format, as well as developing particular intervention programs based on those results. Especially, text and image integration learning programs should be developed for image-dominant readers to better train children in understanding nonlinear narratives.

## 5 Conclusion

This study employed eye-tracking technology to investigate how 6-7-year-old children read nonlinear narrative picture books. Four key findings emerged: (1) children exhibited a predominant “image-first” reading pattern, though deeper cognitive processing occurred during text fixations; (2) path consistency and cross-AOI scanning frequency increased continuously with age; (3) cross-AOI scanning frequency was the strongest predictor of comprehension performance; and (4) three distinct reader types were identified, with integrative readers achieving the highest comprehension. These findings extend text-image integration theory to nonlinear narrative contexts. For picture book designers, the results support age-adaptive visual guidance systems. For educators, the identification of distinct reading patterns suggests differentiated instruction strategies, particularly for image-dominant readers. Understanding these cognitive skills becomes increasingly important as children encounter more nonlinear information structures in digital environments.

## Data Availability

The raw data supporting the conclusions of this article will be made available by the authors, without undue reservation.

## Supplementary material

SUPPLEMENTARY FIGURE S1 Experimental setup of the eye-tracking session, illustrating the Tobii Pro Spectrum eye-tracker positioning, the 24-inch stimulus screen, and the seating arrangement of the child participant at a viewing distance of 60–65 cm.
