# Supplementary figures and images for: Visual guidance patterns and comprehension mechanisms in children’s nonlinear picture book reading: an eye-tracking study
Source: Front Psychol. 2026 Jun 17;17:1755341. doi: 10.3389/fpsyg.2026.1755341 (PMC13321190; doi:10.3389/fpsyg.2026.1755341)

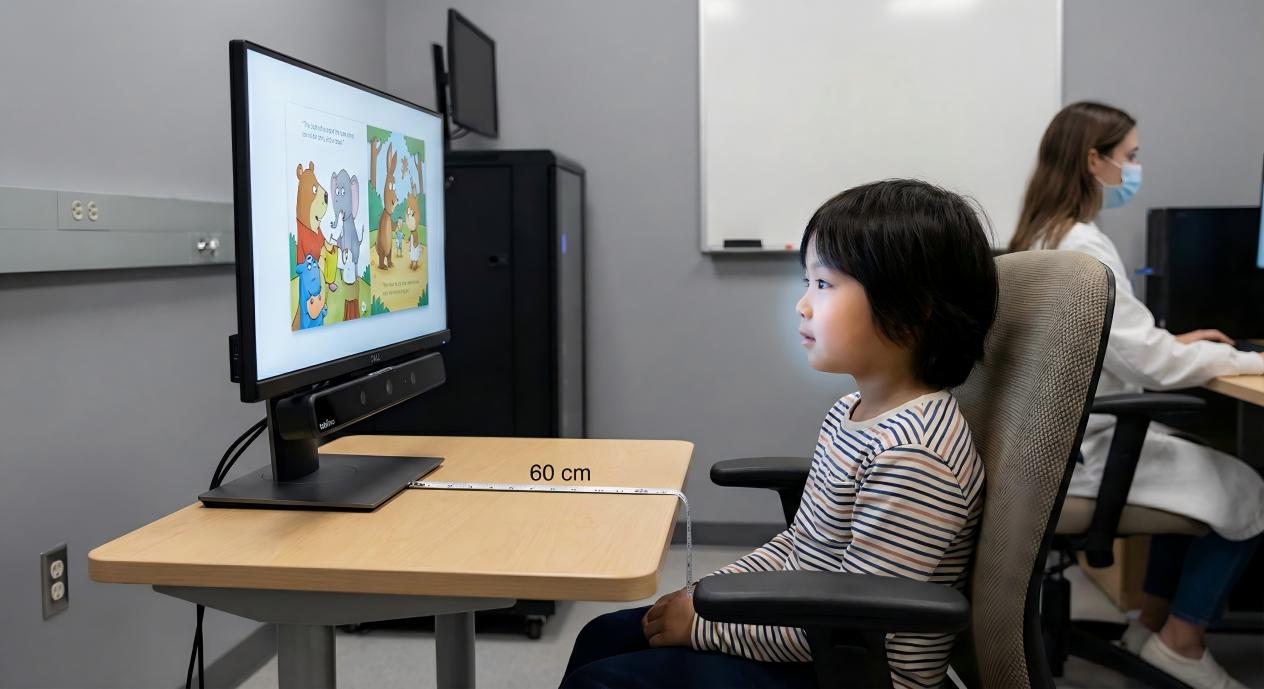


**Supplementary Figure S1. Experimental setup for eye-tracking data collection.**

Supplement: SUPPLEMENTARY FIGURE S1 — Experimental setup of the eye-tracking session, illustrating the Tobii Pro Spectrum eye-tracker positioning, the 24-inch stimulus screen, and the seating arrangement of the child participant at a viewing distance of 60–65 cm. [file Data_Sheet_1.DOCX]
